# Combined anterior and posterior scleritis associated with central retinal vein occlusion: a case report

**DOI:** 10.1007/s12348-012-0066-x

**Published:** 2012-03-13

**Authors:** Vivek Pravin Dave, Annie Mathai, Amit Gupta

**Affiliations:** 1 Smt. Kanuri Santhamma Retina Vitreous Service, L.V.Prasad Eye Institute, Kallam Anji Reddy Campus, Banjara Hills, Hyderabad, 500 034 India; 2 Smt. Kannuri Santhamma Centre for Vitreoretinal Diseases, L.V.Prasad Eye Institute, Education Centre 4th Floor, Kallam Anji Reddy Campus, Banjara Hills, Hyderabad, 500 034 India

**Keywords:** Posterior scleritis, Anterior scleritis, Exudative retinal detachment, Vascular occlusion

## Abstract

**Purpose:**

The purpose of this study is to report an uncommon presentation of anterior and posterior scleritis with central retinal vein occlusion

**Methods:**

We report a 30-year-old female presenting with unilateral anterior and posterior scleritis with concurrent central retinal vein occlusion, the subsequent work-up, and the management. The patient presented with decreased vision and extraocular and intraocular inflammatory signs in the left eye.

**Results:**

At presentation, the best corrected visual acuity in the right eye (OD) was 20/20 and left eye (OS) was perception of light, with inaccurate projection of rays in all quadrants. Intraocular pressure was 12 mmHg in both eyes. OS showed mild proptosis with lid edema. Ocular movements were free and full in both eyes. The bulbar conjunctiva showed nodular anterior scleritis. OS showed mild vitreous haze with an exudative detachment at the posterior pole, disc edema with dilated, congested and tortuous veins and multiple dot blot hemorrhages, flame-shaped hemorrhages, and soft exudates throughout the posterior pole and mid-periphery An ultrasound B scan showed a large hypoechoic area in the sub-Tenon’s space (T-sign) suggestive of periocular fluid collection and thickened sclero–choroidal complex. Orbital ultrasound did not show evidence of any orbital mass or any increase in extraocular muscle thickness. Fundus fluorescein angiography showed few areas of pinpoint hyperfluorescence in the early phase with leakage in the late phase, leakage from the optic disc and vascular staining and pooling of dye in areas of exudative detachment in the late phases in the left eye. Systemic work-up was within normal limits. The patient responded well over the next month with systemic and topical steroids showing complete resolution of the scleritis and exudative retinal detachment.

**Conclusion:**

Simultaneous anterior and posterior scleritis with concurrent central retinal vein occlusion is a rare entity requiring prompt diagnosis and systemic work-up for efficient management

## Introduction

Posterior scleritis is seen commonly in middle-aged females, associated with mild ocular congestion, globe tenderness, and varying amount of vision loss. Overall the age distribution is wide with the youngest case reported at 8 years of age and the oldest at 87 years [1]. Posterior scleritis can present in various ways mimicking orbital tumors, orbital inflammation, optic neuritis, and vasculitis [1–3].Due to its protean manifestations, it is among the most underdiagnosed conditions in ophthalmology.

## Case report

**Fig. 1:**
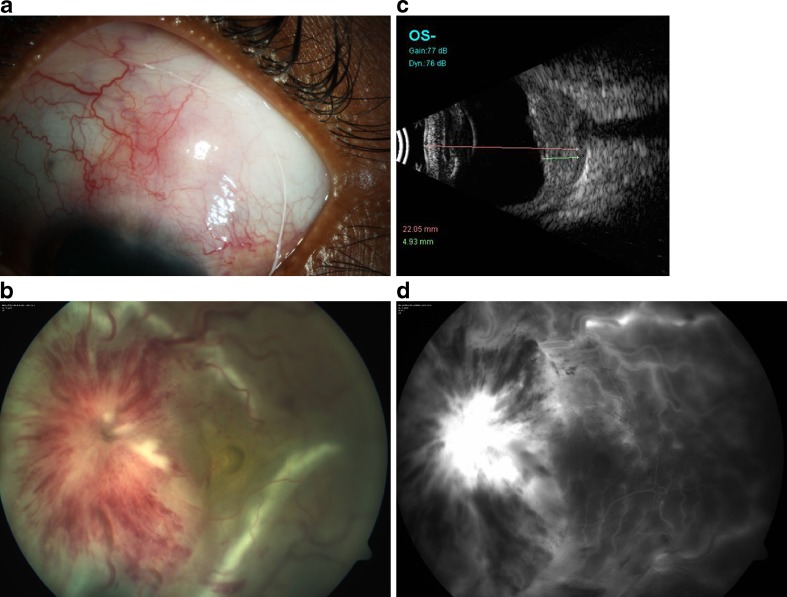
**a** Anterior scleritis at presentation. **b** Fundus photo at presentation showing severe disc edema, peripapillary hemorrhages, retinal hemorrhages, and exudative retinal detachment. **c** Ultrasound B scan at presentation showing thickened sclera and choroid with a “T” sign. **d** Fluorescein angiogram at presentation showing disc and vascular leakage with vascular staining and pin point leaks at the posterior pole

A 30-year-old female patient presented to us with complaints of decreased vision in the left eye since 4 weeks with associated swelling, redness, and watering of the eye. A detailed history ruled out ocular trauma or surgeries or similar episodes in the past in either eye. The patient had no systemic complaints. At presentation the best-corrected visual acuity in the right eye (OD) was 20/20 and left eye (OS) was perception of light, with inaccurate projection of rays (PR) in all quadrants. Intraocular pressure was 12 mmHg in both eyes. OS showed mild proptosis with lid edema. Ocular movements were free and full in both eyes. The bulbar conjunctiva showed ciliary congestion in OS associated with a tender violaceous nodule close to the 1 o'clock limbus with hyperemic deep episcleral plexus suggestive of nodular anterior scleritis (Fig. 1a). Anterior chamber showed 2+ cells and 1+ flare. There were no synechiae. OS pupil showed a gross relative afferent pupillary defect. The lenses were clear in both eyes. The fundus in OD was within normal limits. OS showed mild vitreous haze with an exudative detachment at the posterior pole; disc edema with dilated, congested and tortuous veins; and multiple dot blot hemorrhages; flame-shaped hemorrhages; and soft exudates throughout the posterior pole and mid-periphery (Fig. 1b). An ultrasound B scan showed a large hypoechoic area in the sub-Tenon's space (T-sign) suggestive of periocular fluid collection and thickened sclero–choroidal complex (Fig. 1c). Orbital ultrasound did not show evidence of any orbital mass or any increase in extraocular muscle thickness. Fundus fluorescein angiography showed few areas of pinpoint hyperfluorescence in the early phase with leakage in the late phase, leakage from the optic disc and vascular staining and pooling of dye in areas of exudative detachment in the late phases in the left eye (Fig. 1d). The arteriovenous transit time was normal. There was no abnormal fluorescence in OD.

**Fig. 2:**
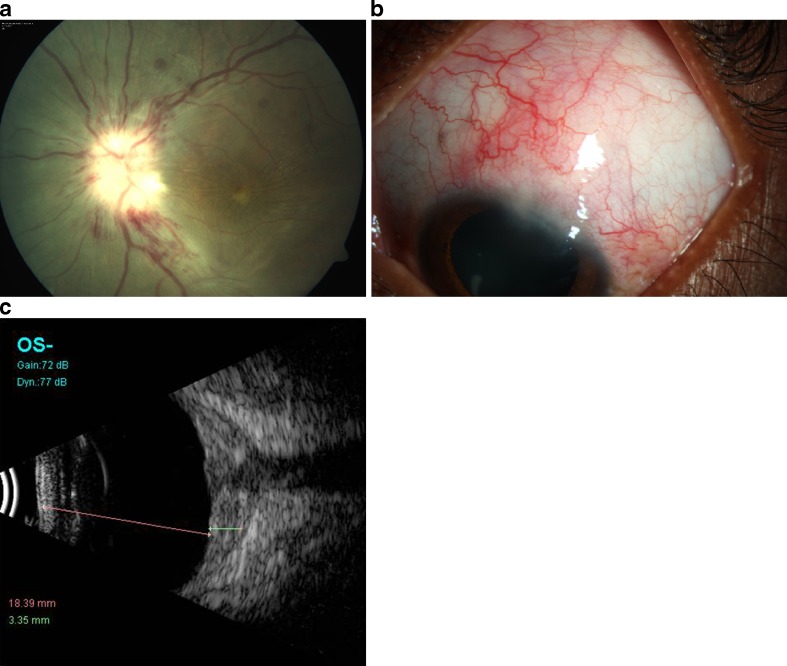
**a** Fundus photo at 1 week posttreatment showing resolving exudative detachment and resolving hemorrhages. **b** Anterior scleritis with mild resolution at 1 week posttreatment. **c** Ultrasound B scan showing decreased thickness of the sclera and choroid and reduction in the “T” sign

**Fig. 3:**
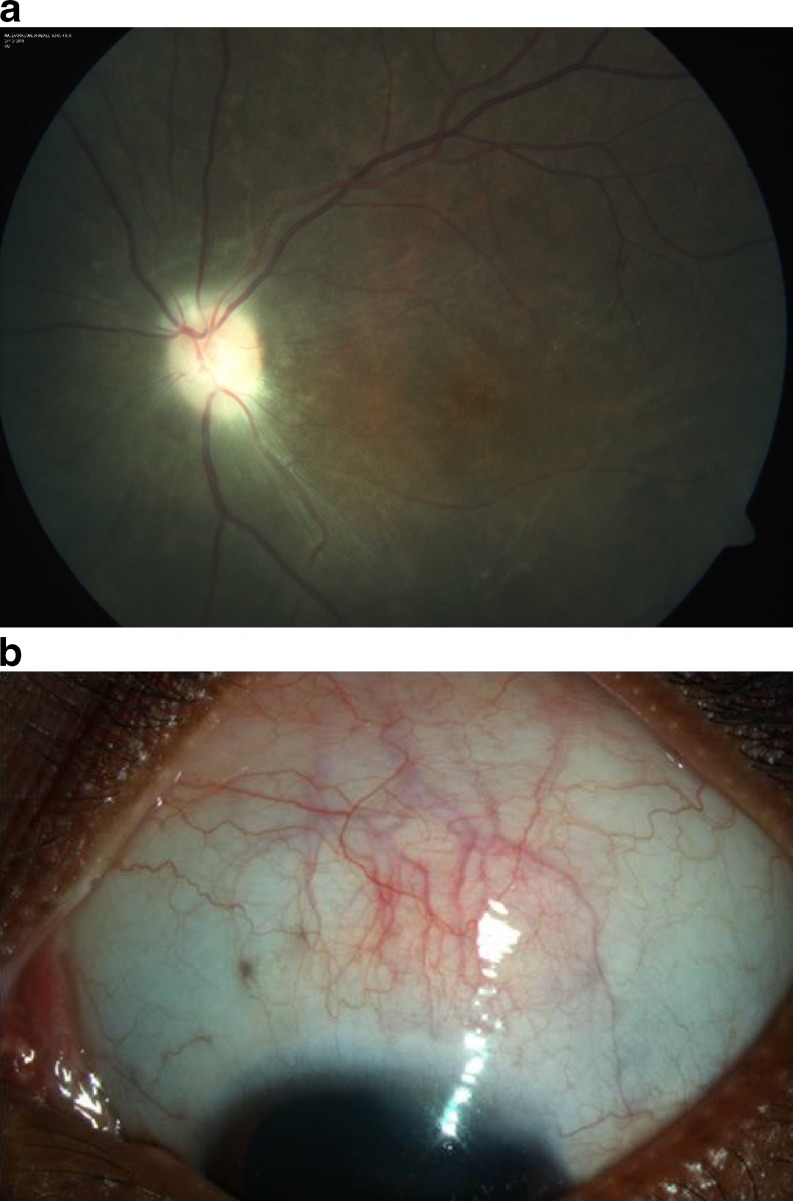
**a** Fundus photo at 1 month posttreatment showing resolved exudative detachment with disc pallor and retinal pigmentary changes. **b** Near-resolved anterior scleritis at 1-month follow-up

This picture was suggestive of combined anterior and posterior scleritis with central retinal vein occlusion. A complete blood count, erythrocyte sedimentation rate, antinuclear antibodies, rheumatoid factor, C-reactive protein, chest X-ray, and sacro iliac joints X-ray were done. All reports were within normal limits. Mantoux test showed a value of 8 mm after 72 h which was within normal range. Cardiac evaluation and a carotid Doppler were done which also were within normal limits. The patient was started on intravenous pulse Methyl prednisolone 500 mg daily for 3 days followed by oral Prednisolone 30 mg (1 mg/kg body weight) tapered over the next month. Topical treatment included eye drops Prednisolone acetate 1 % six times per day and eye drops homatropine 2 % three times per day tapered over the next month. Over 1 week to 1 month posttreatment, the lid edema and proptosis had regressed, the ciliary congestion was markedly reduced. The visual acuity in OS was counting fingers at 1 m with accurate PR. The anterior scleritis had resolved. The retina showed complete resolution of exudative detachment with pigmentary changes over the posterior pole, attenuated vessels, and significant disc pallor with few residual hemorrhages. The ultrasound B scan showed regression of the T-sign and decrease in choroidal thickness (Figs. 2 and 3).

## Discussion

In this case, the patient presented with anterior scleritis, anterior chamber, and vitreous cells, exudative retinal detachment over the posterior pole with multiple peripapillary and retinal dot blot and flame-shaped hemorrhages. The findings were consistent with combined anterior and posterior scleritis with an associated central retinal vein occlusion. Accordingly, we looked for evidence of underlying collagen vascular disorders and systemic vasculitides. The absence of joint pains, fever, skin rashes, evidence of chronic uveitis, and a negative serology ruled out collagen vascular disorders and systemic vasculitides. A normal systemic examination, normal peripheral blood smear, and coagulation profile made leukemia unlikely.

Differentiating orbital inflammatory syndrome from posterior scleritis is difficult as both can have orbital signs and both are exquisitely sensitive to systemic steroids. Intraocular inflammation, though, is not common in orbital inflammatory syndrome [4]. It has been hypothesized on the basis of histopathologic studies that the inflammation in posterior scleritis can contiguously spread to the retinal artery and vein and cause vasculitis with subsequent occlusion [5]. Shukla et al. have described a case of combined central retinal artery and vein occlusion in a case of posterior scleritis [6]. In their case, the anterior segment examination revealed no evidence of anterior scleritis and the systemic work-up was within normal limits. Similar to their case, our case too was treated with intravenous pulse methyl prednisolone followed by tapering doses of oral prednisolone with resolution of the exudative detachment and residual disc pallor with attenuated vasculature. Our case shows that involvement of a particular structure in the eye can sometimes involve an adjacent structure due to contiguous spread of pathology. This can give a mixed clinical picture with overlapping features of multiple conditions. A detailed clinical examination with pathology, physiology, and local anatomy kept in mind can help arrive at the right diagnosis and administer timely treatment. Visual improvement may however be limited depending on the extent of disease involvement.

Though vascular occlusion due to posterior scleritis is mentioned in literature [5–8] it is relatively rare and to the best of our knowledge this is the first report of combined anterior and posterior scleritis with associated central retinal vein occlusion.
